# Heat tolerance classification criteria require population‐specific thresholds for accurate assessment of acclimation state in adults

**DOI:** 10.14814/phy2.70745

**Published:** 2026-02-15

**Authors:** Jacob S. Bowie, Michael R. Szymanski, Jeb F. Struder, Erica M. Filep, Margaret C. Morrissey‐Basler, Gabrielle J. Brewer, Staci N. Thornton, Kyle J. Mahoney, Yasuki Sekiguchi, Oh Sung Kwon, Ki Chon, Douglas J. Casa, Elaine C. Lee

**Affiliations:** ^1^ Department of Kinesiology University of Connecticut Storrs Connecticut USA; ^2^ Biomedical Engineering, College of Engineering University of Connecticut Storrs Connecticut USA; ^3^ Korey Stringer Institute University of Connecticut Storrs Connecticut USA; ^4^ Present address: Department of Exercise and Sport Science University of North Carolina at Chapel Hill Chapel Hill North Carolina USA; ^5^ Present address: Department of Kinesiology and Military Science Texas A&M University‐Corpus Christi Corpus Christi Texas USA; ^6^ Present address: Department of Health Sciences Providence College Providence Rhode Island USA; ^7^ Present address: Sports Performance Laboratory, Department of Kinesiology and Sport Management Texas Tech University Lubbock Texas USA

**Keywords:** heat acclimation, heat tolerance testing, return‐to‐activity, sex differences, thermoregulation

## Abstract

Heat tolerance testing (HTT) assesses responses to heat stress with rectal temperature (T_rec_) and heart rate (HR) thresholds defining individuals as heat‐tolerant (HT) or heat‐intolerant (HI). To evaluate classification criteria by acclimation state and sex. Forty participants (19M/21F, mean ± SD, 23 ± 4 years) completed an HTT (120 min, 5 km·h^−1^, 2% grade) before (PreHA) and after (PostHA) 5 days of repeated exercise in (40°C, 40% RH) categorized as isothermal (exercise intensity adjusted to maintain T_rec_ within 38.5°C–39.5°C, 60 min) exercise‐heat acclimation (HA). Females had lower body surface area (vs. males) (1.7 ± 0.1 vs. 2.0 ± 0.2 m^2^, *p* < 0.001), mass (59.5 ± 5.4 vs. 79.5 ± 10.1 kg, *p* < 0.001), height (164.2 ± 5.7 vs. 179.1 ± 7.6 cm, *p* < 0.001), maximal oxygen consumption (V̇O_2_max, 44.5 ± 5.1 vs. 51.5 ± 5.7 mL·min^−1^·kg^−1^, *p* < 0.001), and velocity at V̇O_2_max (12.9 ± 1.5 vs. 15.0 ± 1.7 km·h^−1^, *p* < 0.001). PreHA 51.1% (mean % HI classification across criteria) of participants were HI (66.7% F/52.6% M), decreasing (*p* < 0.001) to 23.6% PostHA (28.6% F/10.5% M), reflected across criteria (*p* < 0.05). The rate of HI based on plateau in T_rec_ during the final 60 min (ΔT_rec (T120–T60)_ ≥ 0.45°C) was similar (*p* = 0.735) PreHA (15% HI) and PostHA (10%), and HA reduced ΔT_rec (T120–T60)_ (0.30 ± 0.19 vs. 0.19 ± 0.20°C, *p* = 0.001), demonstrating sensitivity to adaptation. HA reduces HI classification rate, but criteria differ in capturing presumed HT and classify more females as HI in naïve and acclimated states.

## Graphical abstract

Heat acclimation status is detected by changes in response to a standard Israeli Defense Force (IDF) heat tolerance test (HTT) intended to evaluate safety among participants returning to duty after exertional heat illness or anticipating exposure to exercise‐heat stress. Measures of heart rate and core temperature during HTT suggest that varying classification criteria differentially designate heat tolerance status among individuals and by sex.

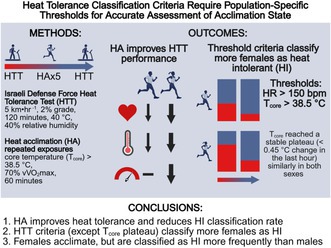

## 1 INTRODUCTION

Risk of exertional heat illness (EHI) in hot environments, or during intense physical activity, is a significant concern in both military and athletic settings. EHIs range from mild dehydration to severe and life‐threatening heat stroke (Bouchama et al., 2022; Casa et al., 2012; Howe & Boden, 2007). To ensure the safety of individuals who have experienced EHI, carefully designed return‐to‐activity (RTA) guidelines are crucial (Roberts et al., 2023). These evidence‐based protocols progressively increase exercise and heat exposure while monitoring physiological responses to avoid reinjury. The protocols serve to verify recovery, assess heat tolerance (HT), facilitate reacclimatization, and prevent EHI recurrence (Roberts et al., 2023).

A critical component of RTA protocols is heat tolerance testing (HTT), which assesses an individual's ability to thermoregulate during exercise in hot conditions. Originating in early research on heat acclimation (HA) to prevent EHI in naive mine workers, HTT has expanded into a variety of sport and occupation‐specific tests (Butler et al., 2023; Dreosti, 1935; Schneider, 2016). In the 1970s, high rates of recurrent heat stroke among post‐EHI soldiers returning to duty prompted the Institute for Military Physiology, Israeli Defense Forces (IDF), to develop a standardized HTT, which evolved into a current clinical standard (Moran et al., 2004, 2007). The IDF HTT is conducted in a climate‐controlled room at 40°C and 40% relative humidity, with participants walking on a treadmill at 5 km·h^−1^ with a 2% grade for 120 min. Core body temperature (typically rectal temperature, T_rec_) and heart rate (HR) are continuously monitored, and HT is determined based on specific T_rec_ and HR thresholds. The IDF HTT has been widely adopted (Butler et al., 2023) and adapted (Heaney, 2017; Heaney et al., 2009) by other military organizations (Alele et al., 2021a) and sports medicine practitioners worldwide, and is referenced as the standard HTT (Roberts et al., 2021; Schermann, Heled, et al., 2018) for assessing HT and guiding RTA decisions following EHI (Koo et al., 2023). HTT is an essential component of RTA monitoring following EHI per the National Athletic Trainers Association (Casa et al., 2013, 2015) and the American College of Sports Medicine (O'Connor et al., 2010) and has been successfully used for post‐EHI RTA in athletes (Rivera‐Brown et al., 2023) and warfighters (Kazman et al., 2013).

While the IDF HTT is commonly used to test males and females, it has several limitations (Druyan et al., 2012). The protocol and physiological thresholds were developed using a dataset of 175 young (18–22 years) male soldiers (Druyan et al., 2013; Ketko et al., 2014; Schermann, Craig, et al., 2018), which may not account for sex‐based differences in physiological responses to heat stress (Druyan et al., 2012). Increased aerobic fitness (Lisman et al., 2014) and acclimation (Mitchell et al., 2021) both improve HTT performance, leading to challenges in determining whether thermoregulatory tolerance is truly being assessed. While HTT provides valuable information about an individual's current HT, association with future EHI risk remains a subject of ongoing research (Schermann, Heled, et al., 2018); data suggest that heat tolerant individuals have reduced rates of recurrent EHS (Kester et al., 2024).

The current study addresses a critical gap in the literature by examining sex‐based differences in HTT classification before and after HA. This research evaluates the need for sex‐specific criteria in HT assessments by comparing physiological responses between females and males during standardized HTTs. Such insights could improve the accuracy and personalization of RTA protocols, improving safety and performance for athletes and warfighters recovering from EHI. Accurate classification is necessary as misclassifying a warfighter as HI after EHI could result in the termination of their military career. In contrast, an HT classification of an HI individual could expose them and their fellow warfighters to excess risk.

## 2 METHODS

### 2.1 Subjects

Healthy participants (*n* = 40, 21F/19M) without a history of EHI (mean ± standard deviation, SD, 23 ± 4 years old) were recruited for the study. All participants were well‐trained (V̇O_2_max > 40/45 mL⋅kg^−1^⋅h^−1^, F/M, respectively) adults (18–50 years) who completed an IDF HTT (120 min at 5 km·h^−1^, 2% grade) before and after 5 days of repeated exercise in the heat (40°C, 40% relative humidity, RH) categorized as isothermic (exercise intensity‐adjusted to maintain T_rec_ at 38.5°C–39.5°C for 60 min) exercise‐HA. All procedures followed the ethical standards of the institutional and national research committee, along with the 1964 Helsinki Declaration and its later amendments or comparable ethical standards. The University of Connecticut Institutional Review Board and the Department of Defense Human Research Protection Office reviewed and approved this study. Written informed consent was obtained from all individual participants included in the study.

### 2.2 Hydration assessment

Before each session, hydration state was confirmed by refractometer measurement of urine specific gravity (USG, Reichert, Lincolnshire, IL, USA) and participants were either cleared for exercise (<1.020), asked to drink 500 mL of water and rechecked (1.020–1.025), or rescheduled if (>1.025, (Casa et al., 2000)). Urine color was recorded using a visual chart following standard procedures (Armstrong et al., 1994).

### 2.3 Study design

Exclusion criteria included self‐reported inability to tolerate exercise in the heat, chronic or acute illness, nontraditional diet, or any other contraindication to exercise and thermoregulation determined by medical staff. During the study, participants were asked to refrain from structured exercise training and from using pain relievers (e.g., acetaminophen, NSAIDs, and aspirin).

Baseline testing included measurement of anthropometric data (i.e., height, body mass, and body composition), hydration, and maximal oxygen consumption (V̇O_2_max, TrueOne 2400, Parvomedics, Salt Lake City, UT, USA). Body surface area (BSA) was estimated with the Mosteller Equation (Mosteller, 1987) from height measured with a wall‐mounted stadiometer (SECA 220, SECA, Hamburg, Germany, ± 2 mm) and body mass (Ohaus Defender 7000 Xtreme, Ohaus Corporation, Parsippany, NJ, USA, ± 0.01 kg). Body composition was assessed by a dual‐energy X‐ray absorptiometry device (Lunar Prodigy Advance system, GE Healthcare, Chicago, IL, USA, RRID: SCR_025004), and region of interest adjustments were performed by a trained research member (GJB). For V̇O_2_max testing specifically, participants jogged on the treadmill at a self‐selected pace (5 min) and then stretched gently (5 min). The test began at a speed equivalent to a jog at a 2% grade and increased incrementally (0.5 or 1 mph) every 2 min until the participant reached volitional fatigue (Green et al., 2023).

HTT and HA occurred in an environmental chamber (CANTROL International Inc. Markham, ON, Canada) designed to maintain precise conditions with temperature (±0.3°C) and humidity control (±3% RH), set to standardized conditions (40°C, 40% RH, wet bulb globe temperature, WBGT ≥32.2°C), recorded every 30 min during and at the end of the session (Kestrel 5400 Heat Stress Tracker, Kestrel Instruments, Nielsen‐Kellerman Co., Boothwyn, PA, USA). Before both HTTs, participants sat in the chamber for an equilibration period (10 min). Testing included the IDF HTT and a time‐to‐temperature test (HTT2, running at 65% vV̇O_2_max, 2% grade, until T_rec_ = 39.5°C). Testing occurred before (PreHA) and after (PostHA) a five‐day isothermic, also referred to as hyperthermic zone (Sekiguchi et al., 2022), HA protocol (participants ran at 70% vV̇O_2_max until T_rec_ reached 38.5°C). From that point on, staff adjusted exercise intensity to maintain T_rec_ between 38.5°C and 39.5°C for 60 min using 30%, 40%, or 70% of vV̇O_2_max. Sessions were separated by a minimum of 24 h and completed within 8 days.

During the IDF HTT, oxygen consumption (V̇O_2_) was measured for 10 min at 30 min and again at 90 min, after which participants were provided 250 mL of water. Metabolic heat production was calculated from V̇O_2_ measurements (Cramer & Jay, 2019). Whole‐body sweat loss (WBSL) was determined by measuring the change in nude body mass from pre‐ to post‐exercise, accounting for fluid intake and urine output (Armstrong et al., 1994). Whole body sweat rate (WBSR) was calculated by dividing WBSL by the total exercise duration. To account for variation in body size, both measures were adjusted for participant BSA (WBSL_BSA_ and WBSR_BSA_).

### 2.4 Cardiovascular and thermoregulatory measurements

HR was continuously monitored using a Polar H10 chest strap monitor (Polar Electro Inc., Lake Success, NY, USA), which uses ECG‐based technology to detect electrical signals from the heart and transmit real‐time heart rate data via Bluetooth to a Polar Vantage M watch (Polar Electro Inc., Lake Success, NY, USA). Data were recorded at exercise start, end, and 5‐min intervals throughout the session, with the watch serving as the intermediate data storage device before subsequent upload to a cloud platform, Polar Flow (Polar Electro Inc., Lake Success, NY, USA), for analysis. T_rec_, measured with a rectal thermistor (YSI 401, Yellow Springs Instruments Inc., Yellow Springs, OH) inserted at least 10 cm past the anal sphincter, was measured continuously with a physiological monitoring system (MP160, BIOPAC Systems Inc., Goleta, CA, RRID: SCR_014829) processed with AcqKnowledge Software (BIOPAC Systems Inc., Goleta, CA, RRID: SCR_014279) and recorded at exercise start, end, and five‐minute intervals throughout the session.

### 2.5 Calculations

We analyzed physiological and environmental data comprehensively to characterize heat adaptation responses, including calculated variables from raw measurements and environmental conditions throughout the protocol. The area under the curve (AUC) was calculated using the trapezoidal method for T_rec_ and HR across all testing sessions, with baseline‐adjusted values for individual differences using the “trapz” function of the *pracma* package (Borchers & Borchers, 2019). To address varied baseline T_rec_, we calculated AUC above physiologically relevant thresholds (37°C, 38°C, and 38.5°C) to quantify thermal strain. Peak and mean values were determined for T_rec_ and HR across each session. We calculated the thermal‐circulatory ratio (TCR) at 60 (T60) and 120 (T120) minutes by dividing T_rec_ by HR (Ketko et al., 2014). The rate of rise (RoR) was calculated as the change from baseline for T_rec_ and exercise start (T0) for HR to end‐exercise divided by exercise duration. Delta values (change from baseline or T0 to end‐exercise or T120) were computed for all primary variables to assess the magnitude of physiological strain. We also calculated the magnitude of change (delta) in all primary variables from PreHA to PostHA to compare adaptation by sex. HA days (1–5) were averaged with HR_AUC_ summed across days.

### 2.6 IDF classification criteria

We classified IDF HTT performance using published criteria (Druyan et al., 2012, 2013; Ketko et al., 2014; Moran et al., 2004, 2007; Schermann, Heled, et al., 2018) summarized elsewhere (Mitchell et al., 2021). While we analyzed all criteria, we excluded the first two iterations of threshold values (Moran et al., 2004, 2007), which were subsequently updated (Druyan et al., 2013). Additionally, we separated the updated criteria into HR, T_rec_, and a plateau in T_rec_ during the last 60 min (ΔT_rec (T120–T60)_) components. PHT values were calculated (https://phtheller.shinyapps.io/HTTest/) with an online calculator, as the algorithm is not publicly available (Schermann, Heled, et al., 2018). The percentage of HI participants was calculated, per threshold, stratified by sex and timepoint (PreHA vs. PostHA). The mean percentage of HI classification was then computed by averaging across all criteria by sex and timepoint (PreHA vs. PostHA).

### 2.7 Statistical analysis

Statistical analyses were conducted using R (version 4.4.1, (R Core Team, 2013)) and RStudio (version 2024.04.2+764) (Allaire, 2012). Data manipulation and analysis were performed using the *tidyverse* suite of packages (Wickham et al., 2019). Data visualization was performed using *ggplot2* (Wickham, 2011), *pheatmap* (Kolde & Kolde, 2015), and *ggpubr* (Kassambara, 2018) packages. Data were assessed for homogeneity of variance using Levene's test, and normality was assessed using the Shapiro–Wilk test and visual inspection of Q‐Q plots (Ott & Longnecker, 2010). Chi‐square tests were used to compare the mean frequency of HI classification PreHA to PostHA for all, females, and males, and by timepoint/group combinations, while Fisher's exact test was applied for criterion‐specific analyses.

For comparisons across timepoints, we performed repeated measures ANOVA (rmANOVA) using the *ez* package to assess main effects of timepoint (within‐subject factor), sex (between‐subject factor), and their interactions, with Greenhouse–Geisser corrections applied when sphericity was violated as determined by Mauchly's test (Lawrence & Lawrence, 2016). For non‐normally distributed repeated measures data, Friedman tests were used for within‐group comparisons across timepoints, while Kruskal–Wallis tests were employed for between‐group comparisons. To assess variation in HR and T_rec_ during the HTT, linear mixed‐effects models were used with subject as a random effect, with time as a continuous variable, and testing interactions between time, timepoint (PreHA vs. PostHA), and sex, with or without inclusion of baseline as a covariate to account for differences in starting values.

Following the rmANOVA, if significant effects were observed, we applied appropriate post hoc tests. For within‐subject comparisons of HA responses (PreHA vs. PostHA), paired *t*‐tests were used for normally distributed data and Wilcoxon signed‐rank tests for non‐normally distributed data for all, females, and males. For normally distributed data with homogeneous variances, independent *t*‐tests were employed for between‐group comparisons; for normally distributed data with unequal variances, Welch's *t*‐tests were used; otherwise, Wilcoxon rank‐sum tests were used. We compared females to males with PreHA to PostHA delta values, pooled values (PreHA and PostHA), and with timepoint‐specific comparisons (PreHA or PostHA). Given the hypothesis‐driven nature of our prespecified physiological comparisons and to balance concerns of Type I and Type II errors in this physiological investigation, we controlled the family‐wise error rate with a Bonferroni correction for only sex‐based comparisons of HA efficacy. A *p* value <0.05 was considered significant, with effect sizes reported for comparisons approaching significance (0.05 < *p* < 0.10). Results are presented as mean ± SD with sex‐specific values (female mean ± SD vs. male mean ± SD, *p* value, effect size). Effect sizes were quantified using Hedges' *g* (Hedges & Olkin, 1985), to correct for the small sample size, with values of 0.2, 0.5, and 0.8 representing small, medium, and large effects, respectively (Cohen, 1988).

## 3 RESULTS

### 3.1 Female and male participants differed in anthropometric and fitness characteristics (Table 1)

**TABLE 1:** Females had lower aerobic fitness and body size and greater body fat than males.

Measure	All (40)	Female (21)	Male (19)	*p* Value	Hedges' *g*
Age (y)	23.2 ± 3.6	24.0 ± 3.7	22.3 ± 3.3	0.115	−0.498
Mass (kg)	69.0 ± 12.8	59.5 ± 5.4	79.5 ± 10.1	**<0.001**	2.464
Lean mass (kg)	52.7 ± 13.1	41.9 ± 4.5	65.3 ± 7.0	**<0.001**	3.941
Fat mass (kg)	14.3 ± 4.4	15.5 ± 3.4	12.9 ± 5.1	0.092	−0.580
Body fat (%)	21.2 ± 7.1	25.8 ± 4.8	15.7 ± 5.3	**<0.001**	−1.973
Height (cm)	171.2 ± 10.0	164.1 ± 5.7	179.0 ± 7.6	**<0.001**	2.189
BSA (m^2^)	1.8 ± 0.2	1.6 ± 0.1	2.0 ± 0.1	**<0.001**	2.723
BMI (kg·m^−2^)	23.5 ± 2.8	21.8 ± 1.9	25.3 ± 2.4	**<0.001**	1.678
Resting heart rate (bpm)	75 ± 13	80 ± 14	70 ± 11	**0.046**	−0.710
V̇O_2_max (mL·min^−1^·kg^−1^)	47.8 ± 6.4	44.5 ± 5.1	51.4 ± 5.7	**<0.001**	1.273
vV̇O_2_max (km·h^−1^)	13.9 ± 1.9	12.9 ± 1.5	15.0 ± 1.7	**<0.001**	1.357

*Note*: Values are mean ± standard deviation. Females and males were compared with Welch's *t*‐test, and the effect sizes were quantified with Hedges' *g* (positive values indicate higher male values). Significant differences (*p* < 0.05) are bold. Body surface area (BSA), body mass index (BMI), velocity at V̇O_2_max (vV̇O_2_max).

Female participants had lower height, body mass, surface area, and lean mass, and higher body fat, trending towards higher fat mass than males. Females had lower V̇O_2_max and vV̇O_2_max, with higher resting HR than males, indicating lower cardiovascular fitness.

### 3.2 Environmental conditions were stable across HA and HTTs

During HA, mean conditions were stable (ambient temperature 39.15 ± 0.14°C, humidity 38.96 ± 0.55 RH%, and WBGT 31.29 ± 0.15°C). For IDF HTT, ambient temperature varied ~0.3°C (PreHA 39.11 ± 0.86 vs. PostHA 39.44 ± 0.88°C, *p* = 0.034, *g* = 0.478) with similar humidity (40.58 ± 3.72 vs. 39.67 ± 5.78 RH%, *p* > 0.05) and WBGT (31.65 ± 1.57 vs. 31.65 ± 0.95°C, *p* > 0.05). HTT2 environmental conditions were similar (ambient temperature 39.03 ± 0.83°C, humidity 39.70 ± 5.84 RH%, and WBGT 31.44 ± 1.82°C, *p* > 0.05).

### 3.3 Exercise‐heat stress caused expected cardiovascular and thermoregulatory strain

During the 5‐day HA protocol, participants averaged (82.8 ± 5.7 min) total exercise time, with (59.7 ± 1.8 min) maintained above the target T_rec_ of 38.5°C. Participants demonstrated expected responses in cardiovascular and thermal strain, shown by elevated HR_mean_ (144 ± 10 bpm), HR_peak_ (180 ± 10 bpm), T_rec mean_ (38.39 ± 0.22°C), and T_rec peak_ (38.95 ± 0.25°C), WBSL (1.67 ± 0.57 L), and WBSR (1.21 ± 0.39 L·h^−1^).

PreHA HTT2 will be treated as an additional exercise–heat exposure for this sub‐investigation, and PostHA HTT2 is excluded. PreHA HTT2 was stressful, HR_peak_ (188 ± 10 bpm), and duration varied (30.7 ± 7.5 min), with 13 (6F/7M) participants ceasing exercise due to volitional fatigue before reaching T_rec_ = 39.5°C (39.29 ± 0.38°C). Given the distinct methodological considerations of this protocol, the analysis will be published separately.

### 3.4 Exercise‐heat stress elicits sex‐specific physiological responses with greater cardiovascular strain in females versus greater thermal and sweat responses in males

During HA, females demonstrated greater cardiovascular strain than males with higher baseline HR, mean values over HA days (1–5), (94 ± 18 vs. 84 ± 12 bpm, *p* < 0.001), HR_mean_ (149 ± 10 vs. 139 ± 10 bpm, *p* < 0.001), and HR_peak_ (182 ± 10 vs. 179 ± 10 bpm, *p* = 0.009). While start and end HR were different, cumulative HR strain, summed across HA days (1–5), was similar in females (4674 ± 866 vs. males 4307 ± 1101 bpm·min, *p* = 0.361). Thermoregulatory responses differed over the HA protocol, females showed lower T_rec peak_ (38.88 ± 0.24 vs. 39.01 ± 0.24°C, *p* < 0.001) and T_rec AUC_ (100.30 ± 22.73 vs. 122.20 ± 25.19°C·min, *p* = 0.007) than males. Females sweat less, WBSL_BSA_ (0.79 ± 0.18 vs. 1.05 ± 0.25 L·m^−2^, *p* < 0.001) and WBSR_BSA_ (0.58 ± 0.14 vs. 0.75 ± 0.17 L·h^−1^·m^−2^, *p* < 0.001), than males. Hydration markers showed females were more hydrated than males at the end of the protocol with lower end‐exercise USG (1.014 ± 0.011 vs. 1.018 ± 0.008, *p* < 0.001) and urine color (3.5 ± 1.7 vs. 4.5 ± 1.7, *p* < 0.001). Collectively, these data reveal sex‐specific physiological strain patterns during exercise‐heat stress: females have elevated HR and lower T_rec_, while males have greater T_rec_ despite greater sweat loss, coupled with pronounced changes in hydration markers.

### 3.5 HA reduces the HI classification rate per established IDF criteria and classifies more females than males as HI

**FIGURE 1:**
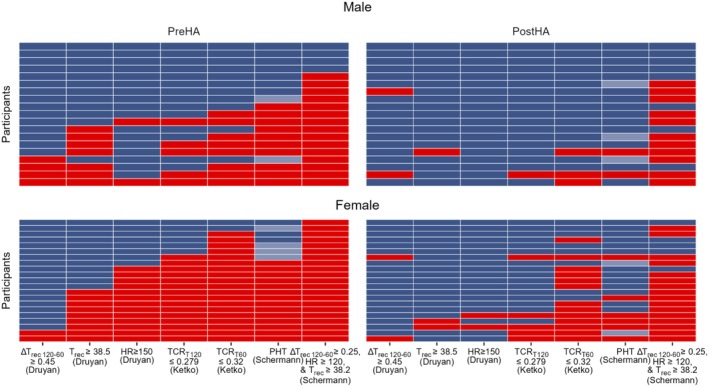
Evidence of a plateau in rectal temperature in the last 60 min (ΔT_rec (T120–T60)_ ≥0.45°C) is the only published Israeli Defense Force (IDF) heat tolerance test criterion that does not classify more females than males as heat intolerant before (PreHA) and after (PostHA) heat acclimation (HA) and does not change with HA. The heatmap displays heat tolerance (dark blue) classification for individual participants (rows) across multiple criteria (columns), where exceeding thresholds classifies participants as heat intolerant (HI, red). HA reduced overall HI classification, from 53.6% PreHA to 25.4% PostHA (χ^2^ = 45.48, *p* < 0.001), with females more frequently classified as HI than males (49.3% F vs. 28.6% M, χ^2^ = 24.30, *p* < 0.001) both PreHA (66.0% F vs. 39.8% M, χ^2^ = 18.14, *p* < 0.001) and PostHA (32.7% F vs. 17.3% M, χ^2^ = 7.91, *p* = 0.005). Among all criteria, only ΔT_rec (T120–T60)_ (Druyan et al., 2013) was unchanged with HA, classifying 15.0% HI PreHA (9.5% F vs. 21.1% M) and 10.0% HI PostHA (9.5% F vs. 10.5% M, *p* = 0.735). In contrast, all other criteria showed significant improvements with HA, including T_rec_ ≥ 38.5°C (Druyan et al., 2013) (40.0%–7.5% HI, *p* = 0.002), heart rate (HR) ≥ 150 bpm (Druyan et al., 2013) (37.5%–5.0% HI, *p* = 0.001), thermal‐circulatory ratio (TCR) at 120 min (TCR_T120_) ≤ 0.279°C·bpm^−1^ (50.0%–15.0% HI, χ^2^ = 9.75, *p* = 0.002), TCR_T60_ ≤ 0.32°C·bpm^−1^ 67.5%–40.0% HI, χ^2^ = 4.18, *p* = 0.041), and probability of heat tolerance (PHT) (75.0%–32.5% HI, χ^2^ = 12.87, *p* < 0.001) (Schermann, Craig, et al., 2018). As IDF criteria were only validated on male data, it follows that those thresholds may misclassify females as HI (Druyan et al., 2012). The PHT algorithm uses the male thresholds, thus inheriting their bias. Borderline heat tolerant (light blue).

HA reduced mean % of HI classification across criteria from PreHA 53.6% to PostHA 25.4% (χ^2^ = 45.48, *p* < 0.001), with more females classified as HI than males (49.3% vs. 28.6%, χ^2^ = 24.30, *p* < 0.001) both PreHA (66.0% vs. 39.8%, χ^2^ = 18.14, *p* < 0.001) and PostHA (32.7% vs. 17.3%, χ^2^ = 7.91, *p* = 0.005, Figure 1). Notably, the rate of classification per the plateau in ΔT_rec (T120–T60)_ ≥ 0.45°C criterion (Druyan et al., 2013) was unchanged by HA, with 15.0% HI PreHA (9.5% F vs. 21.1% M) and 10.0% HI (*p* = 0.735) PostHA (9.5% F vs. 10.5% M). In contrast, HA decreased the rate of HI classification (*p* < 0.05) for all other criteria. The T_rec_ ≥ 38.5°C (Druyan et al., 2013) criterion's rate decreased from 40.0% (42.9% F vs. 36.8% M) to 7.5% (*p* = 0.002) HI (9.5% F vs. 5.3% M), HR ≥150 bpm (Druyan et al., 2013) criterion's rate decreased from 37.5% (61.9% F vs. 10.5% M) to 5.0% (*p* = 0.001) HI (9.5% F vs. 0.0% M), TCR_T120_ ≤ 0.279°C·bpm^−1^ (Ketko et al., 2014) decreased from 50.0% (71.4% F vs. 26.3% M) to 15.0% (*p* = 0.025) HI (23.8% F vs. 5.3% M), the TCR_T60_ ≤ 0.32°C·bpm^−1^ (Ketko et al., 2014) criterion's rate decreased from 67.5% (90.5% F vs. 42.1% M) to 40.0% (*p* = 0.002) HI (61.9% F vs. 15.8% M), the probability of heat tolerance (PHT, (Schermann, Craig, et al., 2018)), including a borderline heat tolerant (BHT) classification grouped with HI for this analysis, criterion's rate decreased from 75.0% (60% HI, 15% BHT) (66.7% F vs. 52.6% M HI, 19% F vs. 10.5% M BHT, 85.7% F vs. 63.2% M total HI) to 32.5% (*p* < 0.001) HI (20% HI, 12.5% BHT) (28.6% F vs. 10.5%M HI, 9.5% F vs. 15.8% M BHT, 38.1% F vs. 26.3% M total HI), and the most stringent, ΔT_rec (T120–T60)_ ≥ 0.25°C and HR ≥120 bpm and T_rec_ ≥ 38.2°C (Schermann, Craig, et al., 2018), criterion's rate decreased from 90.0% (100% F vs. 78.9% M, *p* = 0.048) to 67.5% (*p* = 0.029) HI (76.2% F vs. 57.9% M). The HR ≥150 bpm criterion showed the greatest sex difference (*p* = 0.001), with dramatic improvement in females from 61.9% to 9.5% HI (*p* = 0.001) compared to already low male values (10.5%–0.0%, *p* = 0.486).

### 3.6 Rectal temperature changes

T_rec_ showed significant main effects of time (*p* < 0.001), timepoint (*p* < 0.001), and sex (*p* = 0.020) with a time × timepoint interaction (*p* < 0.001), indicating HA changes the rate of rise. Sex differences in T_rec_ were reduced (*p* = 0.088) when adjusted for baseline values.

#### 3.6.1 HA reduced baseline and T0 T_rec_

**FIGURE 2:**
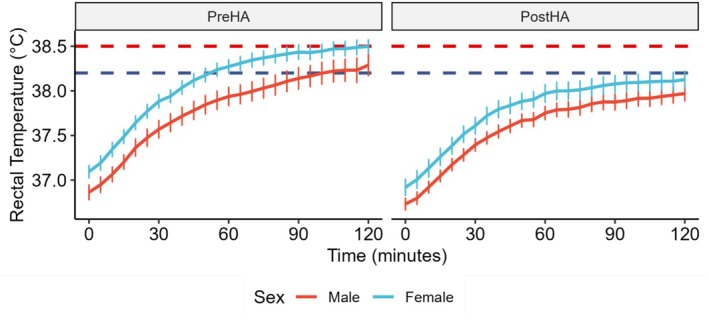
HA reduced rectal temperature (T_rec_) in females (*n* = 21) and males (*n* = 19). HA reduced T_rec_ (*p* < 0.001) with a time × timepoint interaction (*p* < 0.001), indicating HA changes the rate of rise. Sex differences in T_rec_ (*p* = 0.020) were reduced (*p* = 0.088) when adjusted for baseline values. Subjects are classified as heat intolerant if T_rec_ ≥ 38.5°C (red dashed line, (Druyan et al., 2013)) and heat tolerant if T_rec_ < 38.2°C (blue dashed line, (Schermann, Craig, et al., 2018)). Means of 5‐min intervals are plotted with error bars indicating the standard error of the mean. Linear mixed‐effects models with Subject as a random effect, incorporating time as a continuous variable, and testing interactions between time, timepoint (PreHA vs. PostHA), and sex were used to assess variation with baseline‐adjusted models including starting values as a covariate to account for individual differences.

HA reduced baseline and T0 T_rec_ (PreHA: 36.92 ± 0.35 vs. PostHA: 36.80 ± 0.40°C, *p* = 0.032) and T_rec T0_ (PreHA: 36.98 ± 0.38 vs. PostHA: 36.83 ± 0.40°C, *p* = 0.010). Pooled (PreHA and PostHA) data showed females to have marginally higher baseline T_rec_ (36.94 ± 0.39 vs. 36.76 ± 0.34°C, *p* = 0.132) and T_rec T0_ (37.01 ± 0.41 vs. 36.80 ± 0.36°C, *p* = 0.068) than males. PreHA, females had marginally elevated baseline T_rec_ (37.01 ± 0.31 vs. 36.82 ± 0.38°C, *p* = 0.364) and T_rec T0_ (37.09 ± 0.35 vs. 36.86 ± 0.39°C, *p* = 0.240, *g* = −0.611) than males. HA reduced these differences, and PostHA baseline T_rec_ (36.88 ± 0.46 vs. 36.71 ± 0.31°C, *p* = 0.760) and T_rec T0_ (36.92 ± 0.45 vs. 36.73 ± 0.32°C, *p* = 0.532) were similar between sexes. While both sexes adapted to HA, when grouped by sex, only female T_rec T0_ (*p* = 0.043) was reduced, resulting in more similar PostHA values (Figure 2).

#### 3.6.2 HA reduced exercise T_rec_ similarly in females and males

HA reduced overall exercise T_rec_ reflected in T_rec T60_ (38.12 ± 0.44 vs. 37.87 ± 0.41°C, *p* < 0.001), T_rec T120_ (38.41 ± 0.47 vs. 38.05 ± 0.43°C, *p* < 0.001), T_rec peak_ (38.43 ± 0.47 vs. 38.10 ± 0.41°C, *p* < 0.001), and T_rec mean_ (37.95 ± 0.41 vs. 37.69 ± 0.38°C, *p* < 0.001). Pooled data (PreHA and PostHA) showed females to have higher T_rec T60_ (38.12 ± 0.43 vs. 37.84 ± 0.40°C, *p* = 0.012), similar T_rec T120_ (38.31 ± 0.45 vs. 38.13 ± 0.50°C, *p* = 0.488), marginally elevated T_rec peak_ (38.36 ± 0.43 vs. 38.16 ± 0.48°C, *p* = 0.208), and higher T_rec mean_ (37.94 ± 0.39 vs. 37.69 ± 0.40°C, *p* = 0.028). PreHA, females had higher T_rec T60_ (38.29 ± 0.35 vs. 37.94 ± 0.45°C, *p* = 0.036) than males, while T_rec mean_ (38.09 ± 0.33 vs. 37.80 ± 0.45°C, *p* = 0.104), T_rec T120_ (38.50 ± 0.37 vs. 38.31 ± 0.56°C, *p* = 0.242), and T_rec peak_ (38.54 ± 0.36 vs. 38.30 ± 0.55°C, *p* = 0.468) were similar to males. PostHA, these differences were reduced with females demonstrating similar T_rec T60_ (37.97 ± 0.46 vs. 37.75 ± 0.33°C, *p* = 0.348), T_rec mean_ (37.79 ± 0.41 vs. 37.59 ± 0.32°C, *p* = 0.342), T_rec T120_ (38.13 ± 0.46 vs. 37.97 ± 0.39°C, *p* = 1), and T_rec peak_ (38.18 ± 0.44 vs. 38.02 ± 0.36°C, *p* = 0.772) to males. When analyzed by sex, HA improved T_rec_ responses similarly in females and males, reflected in T_rec T120_ (*p* = 0.004 vs. 0.028), T_rec peak_ (*p* = 0.004 vs. 0.024), and T_rec mean_ (*p* = 0.004 vs. 0.048), but not T_rec T60_ (*p* = 0.004 vs. 0.116) where male changes were not significant. Females demonstrate greater adaptation to HA, and PostHA responses are similar to males.

#### 3.6.3 HA reduced ΔT_rec_ and T_rec RoR_ with no differences between sexes

HA reduced the magnitude of T_rec_ elevation from T0 to T120 (ΔT_rec_, 1.49 ± 0.36 vs. 1.26 ± 0.39°C, *p* < 0.001) in females (*p* = 0.036), but not males (*p* = 0.108) with no difference between females and males (PreHA (1.49 ± 0.30 vs. 1.49 ± 0.42°C, *p* = 1) and PostHA (1.25 ± 0.46 vs. 1.26 ± 0.30°C, *p* = 1)). HA reduced T_rec RoR_ (0.012 ± 0.003 to 0.010 ± 0.003°C·min^−1^, *p* < 0.001) with no difference between sexes PreHA (0.012 ± 0.004 vs. 0.012 ± 0.002°C·min^−1^, *p* = 1) or PostHA (−0.011 ± 0.003 vs. −0.010 ± 0.004°C·min^−1^, *p* = 1). Overall, HA reduced ΔT_rec_ and T_rec RoR_ in both sexes with no sex differences despite higher baseline T_rec_ in females.

#### 3.6.4 HA reduced thermal strain, but higher baseline T_rec_ in females drove greater hyperthermic strain

HA reduced the T_rec_ elevation above baseline T_rec AUC_ (23.17 ± 5.58 vs. 20.92 ± 5.76°C·min, *p* = 0.031) and 37°C (T_rec AUC 37_, 23.01 ± 9.38 vs. 17.44 ± 8.60°C·min, *p* < 0.001). T_rec AUC_ did not differ by sex PreHA (23.67 ± 4.62 vs. 22.61 ± 6.57°C·min, *p* = 1) or PostHA (21.07 ± 6.47 vs. 20.75 ± 5.04°C·min, *p* = 1). HA did not change T_rec AUC_ in males (*p* = 0.800) or females (*p* = 0.360). T_rec AUC 37_ was marginally elevated in females PreHA (26.00 ± 7.67 vs. 19.86 ± 10.22°C·min, *p* = 0.160) and PostHA (19.65 ± 9.28 vs. 15.00 ± 7.23°C·min, *p* = 0.336). Overall, HA reduced thermal strain, while females maintained higher T_rec AUC 37_ than males, both PreHA and PostHA, due to higher baseline T_rec_, indicating a similar change from baseline, but more time spent at higher temperatures.

#### 3.6.5 HA reduced ΔT_rec_ _(T120–T60)_, indicating a more stable plateau

**FIGURE 3:**
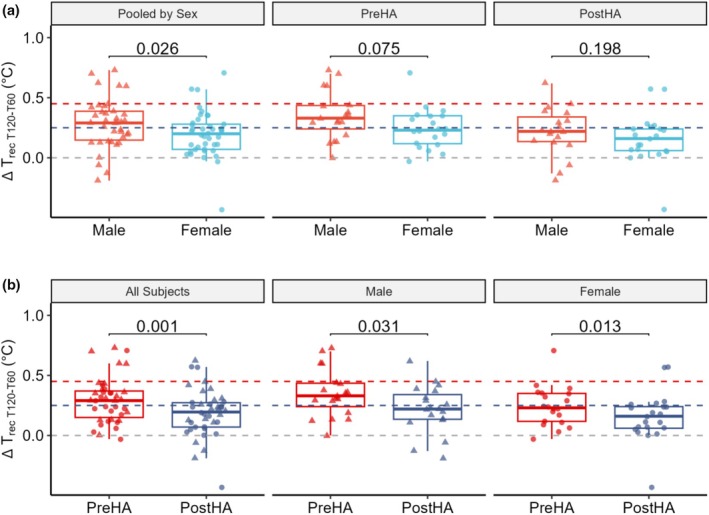
Heat acclimation (HA) decreased the change in rectal temperature in the last 60 min (ΔT_rec (T120–T60)_) with females trending lower than males before (PreHA) but not after (PostHA). Data are presented for all participants combined (ALL, *n* = 40), by sex (Female, *n* = 21; Male, *n* = 19), and by timepoint PreHA, PostHA, and pooled (PreHA and PostHA combined) with individual data points overlaid. Paired *t*‐tests were used to compare PreHA versus PostHA conditions within each group, and independent *t*‐tests were used to compare between groups, with *p* values above comparison bars. Dashed lines indicate thresholds classifying subjects as heat intolerant ≥0.45°C (red, (Druyan et al., 2013)) or heat tolerant <0.25°C (blue, (Schermann, Craig, et al., 2018)) with zero (gray) included to illustrate decreases.

HA reduced ΔT_rec (T120–T60)_ (0.30 ± 0.19 vs. 0.19 ± 0.20°C, *p* = 0.001). Females had similar ΔT_rec (T120–T60)_ as males (Pooled: 0.20 ± 0.19 vs. 0.29 ± 0.21°C, *p* = 0.104), and these differences were not significant when analyzed per timepoint PreHA (0.24 ± 0.17°C vs. 0.35 ± 0.20°C, *p* = 0.300) or PostHA (0.16 ± 0.21 vs. 0.22 ± 0.20°C, *p* = 0.792), with significant reductions in both females (*p* = 0.052) and males (*p* = 0.124, Figure 3).

### 3.7 Heart rate changes

HR responses during heat tolerance testing showed significant main effects of time (*p* < 0.001), timepoint (*p* < 0.001), and sex (*p* < 0.001). HA caused greater HR reductions in females (time × timepoint × sex interaction: *p* = 0.040).

#### 3.7.1 HA reduced T0 HR in females but not males

HA slightly reduced T0 HR in females (96 ± 17 vs. 89 ± 18 bpm, *p* = 0.144) but not males (82 ± 10 vs. 85 ± 10 bpm, *p* = 1), resulting in no change in pooled data (89 ± 16 vs. 87 ± 15 bpm, *p* = 0.892), with females demonstrating a reduction while males did not (−7 ± 14 vs. 2 ± 10 bpm, *p* = 0.004). Females had greater HR_T0_ than males (92 ± 18 vs. 84 ± 10, *p* = 0.028) PreHA (96 ± 17 vs. 82 ± 10, *p* = 0.005), but not PostHA (89 ± 18 vs. 85 ± 10, *p* = 1).

#### 3.7.2 HA reduced HR during the HTT more in females than males, and female HR was higher than males

**FIGURE 4:**
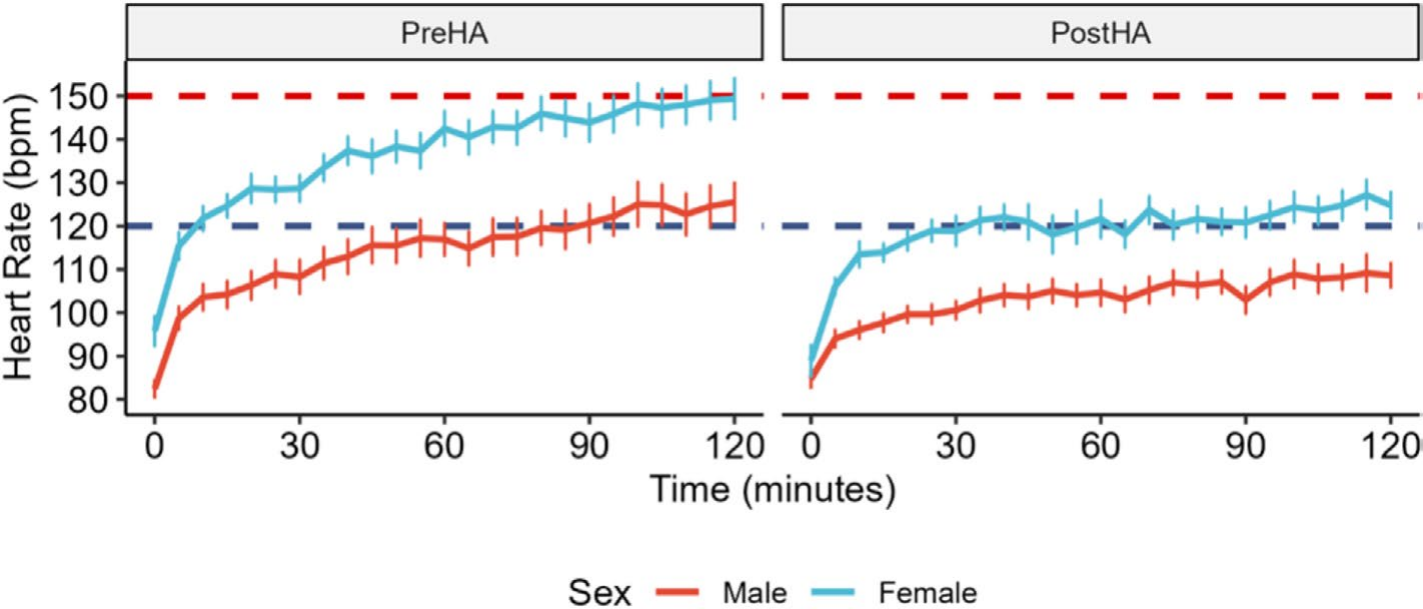
Heat acclimation (HA) reduced heart rate (HR) during heat tolerance testing in females (*n* = 21) and males (*n* = 19). HR responses during heat tolerance testing showed significant main effects of time (*p* < 0.001), timepoint (*p* < 0.001), and sex (*p* < 0.001). Females had higher HR than males, both PreHA and PostHA (*p* < 0.001). HA caused greater HR reductions in females (time×timepoint×sex interaction: *p* = 0.040). Means of five‐minute intervals are plotted with error bars indicating the standard error of the mean. Linear mixed‐effects models with subject as a random effect, incorporating time as a continuous variable, and testing interactions between time, timepoint (PreHA vs. PostHA), and sex were used to assess variation with baseline‐adjusted models including starting values as a covariate to account for individual differences. Subjects were classified as heat intolerant if HR ≥150 bpm (red dashed line, (Druyan et al., 2013)), and heat tolerant if HR < 120 bpm (blue dashed line, (Schermann, Craig, et al., 2018)).

HA reduced exercise HR (HR_T60_,130 ± 22 vs. 114 ± 19 bpm, *p* < 0.001), HR_T120_ (138 ± 24 vs. 117 ± 17 bpm, *p* < 0.001), HR_mean_ (126 ± 19 vs. 111 ± 14 bpm, *p* < 0.001), and HR_peak_ (143 ± 23 vs. 125 ± 19 bpm, *p* < 0.001). Females demonstrated greater reductions than males in HR_T60_ (−21 ± 17 vs. −12 ± 9 bpm, *p* = 0.032), HR_T120_ (−26 ± 16 vs. −17 ± 13 bpm, *p* = 0.036), HR_mean_ (−18 ± 8 vs. −11 ± 9 bpm, *p* = 0.004), but not HR_peak_ (−21 ± 13 vs. −15 ± 12 bpm, *p* = 0.216). PreHA, females had higher HR_T60_ (143 ± 19 vs. 117 ± 16 bpm, *p* < 0.001), HR_T120_ (149 ± 22 vs. 126 ± 21 bpm, *p* = 0.004), HR_mean_ (137 ± 15 vs. 114 ± 16 bpm, *p* < 0.001), and HR_peak_ (155 ± 18 vs. 130 ± 20 bpm, *p* < 0.001) than males. While the difference was reduced, HR remained elevated in females PostHA with higher HR_T60_ (122 ± 20 vs. 105 ± 13 bpm, *p* = 0.012), HR_T120_ (125 ± 16 vs. 109 ± 14 bpm, *p* = 0.004), HR_mean_ (119 ± 3 vs. 103 ± 11 bpm, *p* < 0.001), and HR_peak_ (134 ± 15 vs. 115 ± 17 bpm, *p* = 0.004) than males (Figure 4).

#### 3.7.3 HA reduced cumulative HR strain and RoR in both sexes, but female values were greater than males PostHA

HA reduced HR_AUC_ (882 ± 410 vs. 598 ± 301 bpm·min, *p* < 0.001), ΔHR (49 ± 21 vs. 30 ± 15 bpm, *p* < 0.001), and HR_RoR_ (0.41 ± 0.18 vs. 0.25 ± 0.12 bpm·min^−1^, *p* < 0.001) similarly between sexes. PreHA, females had slightly elevated HR_AUC_ (980 ± 409 vs. 774 ± 393 bpm·min, *p* = 0.113), ΔHR (54 ± 22 vs. 43 ± 19 bpm, *p* = 0.106), and HR_RoR_ (0.45 ± 0.18 vs. 0.36 ± 0.16 bpm·min^−1^, *p* = 0.106) vs. males. PostHA, female values were much greater than males with HR_AUC_ (733 ± 291 vs. 449 ± 241 bpm·min, *p* = 0.008), ΔHR (36 ± 15 vs. 24 ± 11 bpm, *p* = 0.032), and HR_RoR_ (0.30 ± 0.13 vs. 0.20 ± 0.09 bpm·min^−1^, *p* = 0.036).

#### 3.7.4 HA increased TCR in both sexes, and females have lower TCR than males (Figure 5)

**FIGURE 5:**
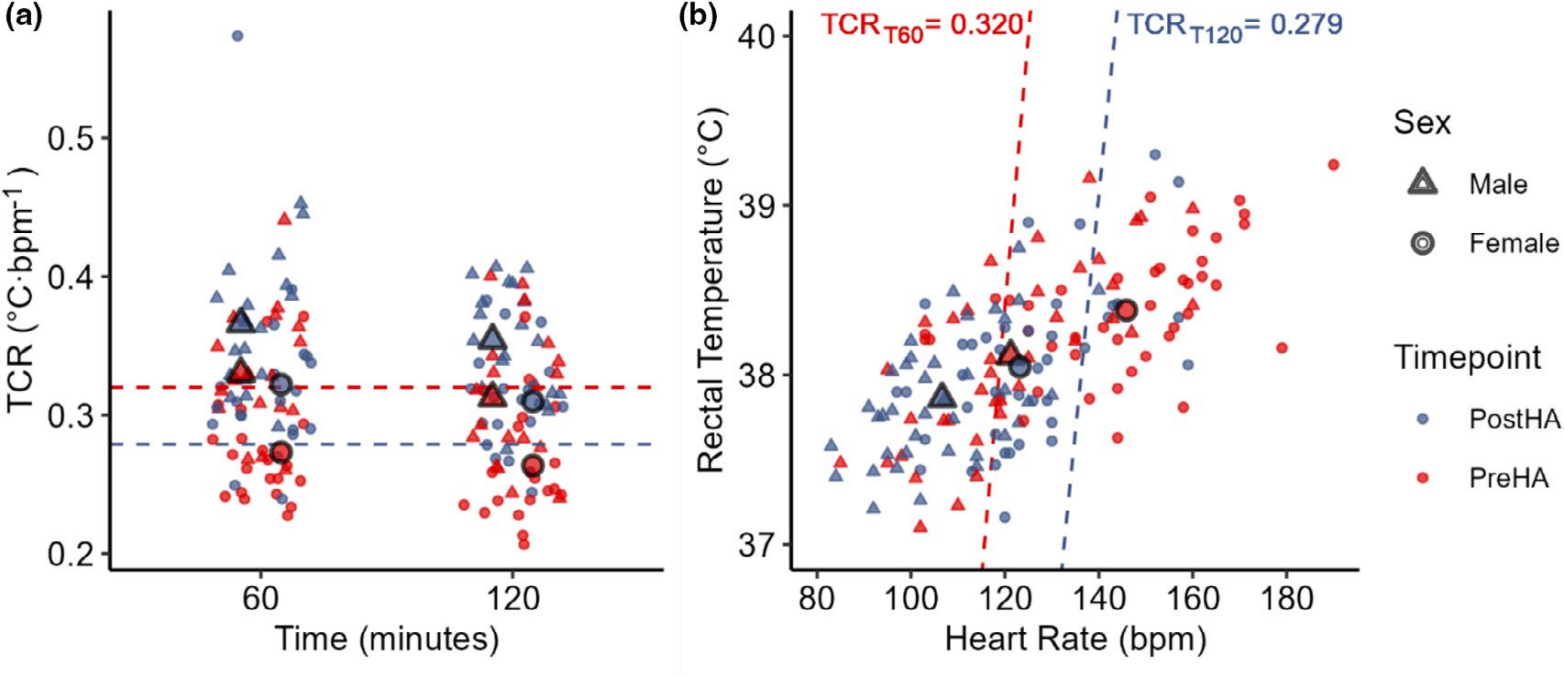
The thermal‐circulatory ratio (TCR) thresholds proposed by Ketko et al. (2014) classify more females than males as heat intolerant, due to elevated rectal temperature (T_rec_) and heart rate (HR), at 60 min (T60) TCR_T60_ ≤ 0.32°C·bpm^−1^ (red dashed line) before heat acclimation (PreHA, 90.5% F vs. 42.1% M, *p* = 0.002) and after (PostHA, 61.9% F vs. 15.8% M, *p* = 0.004), and 120 min (T120) TCR_T120_ ≤ 0.279°C·bpm^−1^ (blue dashed line, PreHA, 71.4% F vs. 26.3% M, *p* = 0.010) and (PostHA, 23.8% F vs. 5.3% M, *p* = 0.186). (a) Females (pooled PreHA and PostHA) have lower TCR_T60_ (0.298 ± 0.05 vs. males 0.348 ± 0.04°C·bpm^−**1**^, *p* < 0.001 and TCR_T120_ (0.287 ± 0.04 vs. males 0.348 ± 0.04°C·bpm^−**1**^, *p* < 0.001). (b) Females have higher T_rec_ than males at T_rec T60_ (38.13 ± 0.43 vs. 37.84 ± 0.40°C, *p* = 0.012), but not at T_rec T120_ (38.31 ± 0.45 vs. 38.14 ± 0.50°C, *p* = 0.388). Females have higher HR than males at HR_T60_ (132 ± 22 vs. 111 ± 16 bpm, *p* < 0.001) and at HR_T120_ (137 ± 23 vs. 117 ± 19 bpm, *p* < 0.001). TCR thresholds were developed for their sensitivity and specificity to classify heat tolerance with data from only male participants and should not be applied to females. Outlined shapes indicate mean values per sex and time point.

HA increased TCR_T60_ (0.301 ± 0.05 vs. 0.343 ± 0.06°C·bpm^−1^, *p* < 0.001) and TCR_T120_ (0.287 ± 0.05 vs. 0.331 ± 0.4°C·bpm^−1^, *p* < 0.001) in females (*p* = 0.024, *p* < 0.001) and males (*p* = 0.068, *p* = 0.024). Females had consistently lower TCR_T60_ (0.298 ± 0.06 vs. 0.348 ± 0.05°C·bpm^−1^, *p* < 0.001) and TCR_T120_ (0.287 ± 0.05 vs. 0.334 ± 0.49°C·bpm^−1^, *p* < 0.001) than males PreHA (TCR_T60_ 0.273 ± 0.04 vs. 0.330 ± 0.04°C·bpm^−1^, *p* < 0.001, TCR_T120_ 0.264 ± 0.042 vs. 0.312 ± 0.05°C·bpm^−1^, *p* = 0.003) and PostHA (TCR_T60_ 0.322 ± 0.07 vs. 0.366 ± 0.05°C·bpm^−1^, *p* = 0.040, TCR_T120_ 0.310 ± 0.04 vs. 0.354 ± 0.04°C·bpm^−1^, *p* < 0.001). Differences in HR, but not T_rec_, drove TCR lower in females.

#### 3.7.5 HA reduced relative exercise intensity in females, but not in males

**FIGURE 6:**
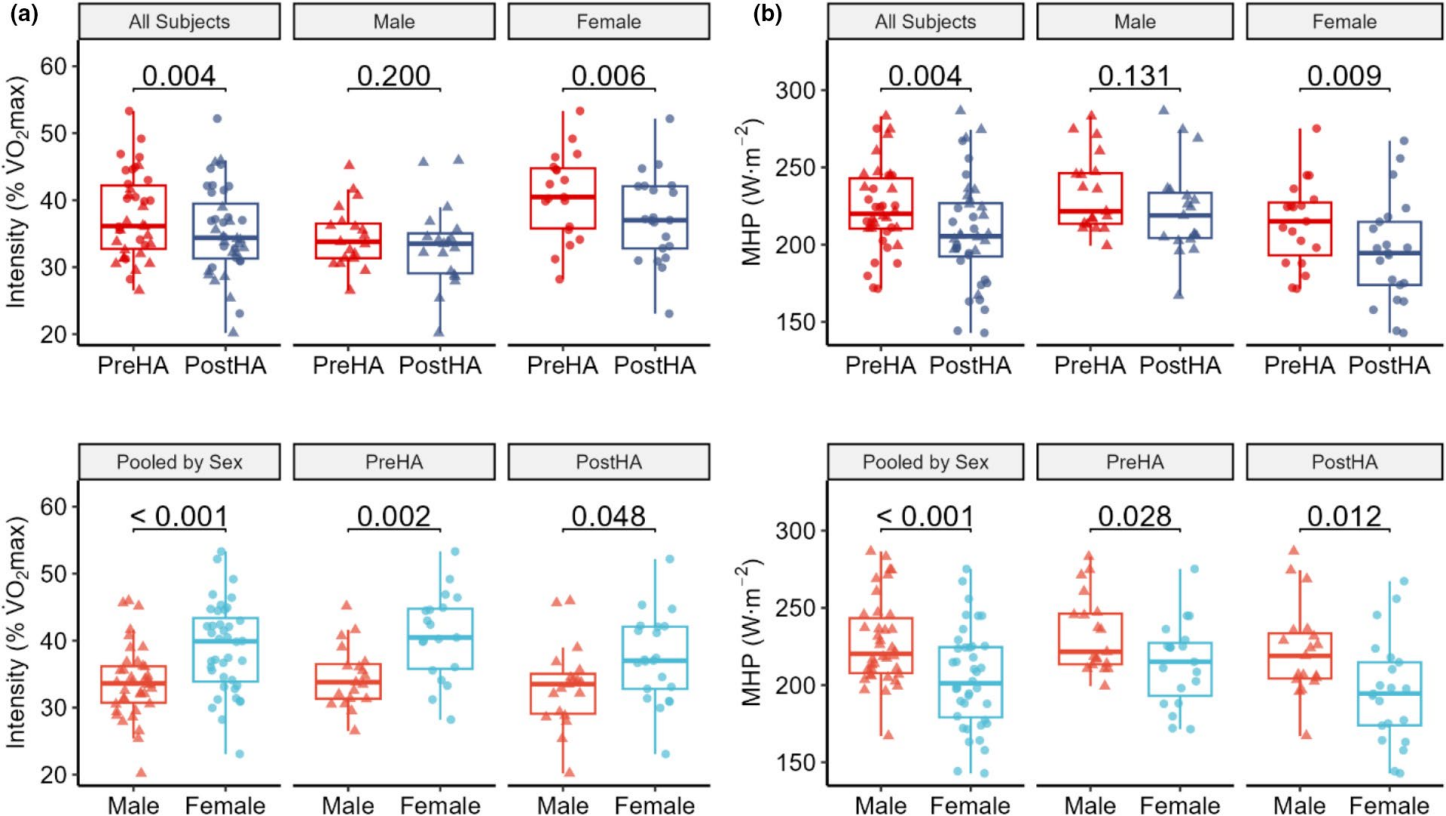
(a) Relative intensity (%VO_2_max) is higher in females than males, and heat acclimation (HA) decreased relative intensity only in females. This reduction from before HA (PreHA) resulted in more similar values after HA (PostHA), but females still worked at a higher relative intensity. (b) Metabolic heat production (MHP) is lower in females than males, and HA reduces MHP in females but not males. MHP is normalized to body surface area and indicates that, despite working at a higher relative intensity, females generate less heat than males. Data are presented for all participants combined (ALL, *n* = 40), by sex (Female, *n* = 21; Male, *n* = 19), and by timepoint PreHA, PostHA, and pooled (PreHA and PostHA combined). Boxplots show the median (center line), interquartile range (box), and 1.5 × interquartile range (whiskers), with individual data points overlaid. Paired *t*‐tests were used to compare PreHA versus PostHA conditions within each group, and independent *t*‐tests were used to compare between groups, with *p* values above comparison bars.

HA reduced relative exercise intensity (37.67 ± 6.36 vs. 35.34 ± 6.64 %V̇O_2_max, *p* = 0.004) in females (40.74 ± 6.46 vs. 37.30 ± 6.56, *p* = 0.024), but not in males (34.60 ± 4.63 vs. 33.89 ± 5.44% V̇O_2_max, *p* = 0.800). Females exercised at a higher intensity than males (38.94 ± 6.66 vs. 33.89 ± 5.44% V̇O_2_max, *p* < 0.001) PreHA (*p* = 0.002) and PostHA (*p* = 0.048). Females demonstrated greater reductions PreHA to PostHA than males (−3.31 ± 4.57 vs. −1.42 ± 4.60% V̇O_2_max, *p* = 0.308) but did not reach statistical significance. Increased relative work rates likely contributed to increased exercise HR (Figure 6).

## 4 DISCUSSION

While the IDF HTT has been used to evaluate HT in athletes and EHI patients, test performance is affected by factors other than thermoregulatory and fitness‐based tolerance or HA status (Mitchell et al., 2021). External factors that improve performance, potentially in the absence of true resilience to combined exercise‐heat stress, include cardiovascular fitness, body size, body fat % (Alele et al., 2021b), and sex (Druyan et al., 2012). We aimed to quantify the differences in HI and HT assessment based on published criteria and data from our studies of fit males and females, finding that HA reduces HI classification rate, but criteria differ in capturing presumed HT PostHA, and disproportionately among females (vs. males) in naïve and acclimated states. Criteria based on reaching a temperature plateau appear to be more robust.

Of note in interpretation, we observed differences in HA between males and females. Females had higher HR and lower T_rec_ than males despite lower sweat rates, even when adjusted for body surface area. During the HTT, baseline T_rec_ was higher in females (vs. males), and although ΔT_rec_ and T_rec AUC_ were similar, females experienced a greater hyperthermic strain, T_rec AUC 37_. HA protocols and target temperatures may need to account for variation in baseline T_rec_ to deliver similar adaptation impulses. Additionally, we observed variability in the environmental conditions that were statistically different but were within the environmental chamber's expected variation (±0.3°C) and support a recommendation to record and report dynamic environmental conditions.

Investigations of the IDF typically use T_rec_ ≥ 38.5°C, ΔT_rec (T120–T60)_ ≥ 0.45°C and HR ≥150 bpm criteria to determine HI, with observed HI rates of 6%–74% (Druyan et al., 2013; Moran et al., 2004, 2007; Northway et al., 2023). Using these three criteria in our presumably tolerant population (no history of EHI, well‐trained participants) PreHA, we found a rate of 55.0% HI (61.9% F vs. 47.4% M). The range of detecting HI individuals is large, and our data are within previously observed rates.

HA is effectively detected with HTT. One investigation of eight repeated IDF HTTs found that HT improved markedly from the first to the eighth session in a criteria‐dependent manner (Mitchell et al., 2021). In 13 male participants with no history of EHI, the ΔT_rec (T120–T60)_ ≥ 0.25°C and HR ≥120 bpm and T_rec_ ≥ 38.2°C (Schermann, Craig, et al., 2018) criteria classified 46.2% HI, improving to 23.1% PostHA. Per this criterion, in males, we found an elevated rate of 78.9% PreHA to 57.9% PostHA. Using the T_rec_ ≥ 38.5°C and HR ≥150 bpm thresholds, the authors classified 15.4% HI PreHA improving to 7.7% PostHA, while we found HI rates of 42.1% PreHA to 5.3% PostHA. One of the factors that may have contributed to the lower HI rates is a two‐fold difference in water intake during the test, with Mitchell et al. providing 200 mL of water every 20 min (1200 mL) versus our protocol, which provided only 250 mL at two timepoints (500 mL). This aspect of our study raises an important consideration for interpretation in the field related to the use of HTT to determine tolerance state without an HA‐induced tolerance as confirmation. Successful HTT resulting in designation as HT reflects a comprehensive performance and ability to tolerate one defined HTT protocol, and the confirmation that HA improves that performance adds confidence to the interpretation of HI or HT.

Few studies of the IDF HTT include females. One study examining HTTs performed during both the luteal and follicular phases found that 56% of females were consistently classified as HI in both phases, with six participants classified as HI during only one phase (Yanovich et al., 2019). This illustrates the limited impact of menstrual cycle phase on HTT outcomes.

A critical aspect of interpretation is the acknowledgment that the IDF uses a set work rate regardless of individual fitness level. We found that females exercised at a higher relative intensity than males during the test, consistent with their lower cardiovascular fitness. The increased relative exercise intensity likely drove HR higher (Mann et al., 2013). This could explain why absolute HR and T_rec_ thresholds, developed using only male data, classified females as HI at higher rates. We demonstrate that females have physiological differences in their response to HTT, which result in their elevated HI classification rate. These differences include a higher baseline and exercise T_rec_ and HR with a clear plateau in T_rec_, indicating the ability to thermoregulate effectively at slightly higher temperatures.

Complicating the interpretation of HT status is the contribution of individual fitness and selection of core temperature and heart rate thresholds interpreted during the HTT. In Yanovich et al.'s 2019 study HT participants had 18% higher V̇O_2_max than HI individuals. This may be associated with reduced HR at the end of HTT, which is our top criterion (HR_T120_) for classifying females as HI in the current study. Another investigation using a more challenging Naval Health Research Center protocol (5.31 km·h^−1^ 4% grade, 40°C 40% RH) assessed 65 (32F/33M) subjects and found 73.8% HI. Heat intolerant individuals had smaller body size (height, weight) and lower V̇O_2_max (45 vs. 52 mL·min^−1^·kg^−^) and heat tolerant individuals had lower body fat % and resting HR (Northway et al., 2023). Of the HI subjects, five‐fold more participants exceeded HR thresholds (*n* = 40, ≥160 bpm) than the T_rec_ threshold (*n* = 8, ≥38.6°C). Primary variables predicting HTT outcome were HR during thermoneutral exercise, V̇O_2_max, and age, illustrating the impact of individual fitness.

Related to core temperature, we observed that individuals classified as HI would likely be classified as HT if ΔT_rec (T120–T60)_ ≥ 0.45°C were used. Two investigations by the Institute of Military Physiology further this point. Researchers studied nine female HTT records and found an elevated rate of HI in females (67% F, *n* = 9 vs. 26% M, *n* = 170) in line with our results (Druyan et al., 2012). A recent publication from the same group used ΔT_rec (T120–T60)_ ≥ 0.45°C criteria and found only 14% (3/21) of females were intolerant, while 6% of males (32/495) were intolerant (Akavian et al., 2025) This supports our conclusion that the ΔT_rec (T120–T60)_ ≥ 0.45°C performed well for both males and females, while HR thresholds classified females as HI at a higher rate than males.

Our data highlights the importance of considering multiple criteria when assessing HT, as different measures may capture varied aspects of physiological responses to heat stress. While the most recent method, PHT, is more nuanced than previous methods, the algorithm still uses factors to penalize the PHT score for T_rec_ > 38.5°C, HR > 150 bpm, and ΔT_rec (T120–T60)_ ≥ 0.45. Using these thresholds likely contributed to the HI scoring of the females. Thus, we recommend practitioners use ΔT_rec (T120–T60)_ when assessing HT in females and males.

Our work reveals significant sex‐based differences in physiological responses to HTTs and HA, highlighting the need for sex‐specific criteria. Females consistently exhibited higher T_rec_ and HR during HTT despite similar relative adaptations to HA. These findings align with previous research demonstrating sex‐specific thermoregulatory responses, potentially due to hormonal influences and anthropometric differences. Interestingly, after HA, females were more similar to males in their response, with differences remaining primarily in HR responses.

### 4.1 Limitations

We did not assess the female menstrual cycle phase before testing, which may change resting and exercise T_rec_ > 0.5°C (Giersch et al., 2020) and may have a slight impact on HTT outcomes (Yanovich et al., 2019). Our participants were young (23 ± 4 years), well‐trained individuals without a history of EHI, so findings may not generalize to older populations, untrained individuals, or those with prior EHI. Our HA protocol was shorter (5 days) than standard (10–14 days) protocols, which may yield different adaptation patterns between sexes (Périard et al., 2015). After the HA protocol, we did not retest V̇O_2_max to determine the training effects of the HA protocol, which could explain relative intensity reductions in females. We did not match female and male participants by body size, composition, or mass. We did not model the impacts of variations in these parameters on HTT outcomes. We do not include expert assessment of HTT outcomes for ground truth HT classification; thus, we could not calculate the sensitivity and specificity of the criteria. Finally, we did not perform follow‐up testing to determine the persistence of adaptations or whether sex differences in HT classification would emerge during the HA decay, which is an avenue for further investigation.

## 5 CONCLUSIONS

This study reveals significant sex‐based differences in physiological responses to HTTs and HA, highlighting the need for sex‐specific criteria. Females consistently exhibited higher T_rec_ and HR during HTT despite similar relative adaptations to HA. These findings align with previous research demonstrating sex‐specific thermoregulatory responses potentially explained by hormonal influences and anthropometric differences. Current HTT criteria, developed using only male subjects, disproportionally classify females as HI. The best‐performing criterion for both sexes was ΔT_rec (T120–T60)_ ≥ 0.45°C. Importantly, females showed greater physiological adaptations after HA than males, though they maintained higher exercise HR. PostHA differences between males and females were reduced. Our findings support implementing sex‐specific thresholds for accurate assessment of return‐to‐activity readiness following heat‐related injuries.

Further work is necessary to recommend female‐specific thresholds for HR and T_rec_ or more nuanced sex‐specific criteria. These may require adjusting thresholds for HT per baseline differences, as with T_rec_, a baseline difference of ~0.2°C may indicate a threshold shift to ≤38.7°C, as there were no differences between males and females in ΔT_rec_. Similar findings are evident in female HR_T0_ ~13 bpm, but ΔHR even at PostHA is elevated ~20 bpm; thus, increasing the threshold to ≤170 bpm for HT status may be appropriate. Further work is necessary to validate the sensitivity and specificity of these thresholds before they can be recommended for use in clinical practice.

## AUTHOR CONTRIBUTIONS

All authors contributed to the study design, data collection, and interpretation, reviewed the manuscript, and approved the final version. JSB and ECL analyzed and interpreted data and drafted the manuscript.

## FUNDING INFORMATION

This study was supported by Department of Defense, Award Number W81XWH‐21‐1‐0558 to DJC and ECL.

## CONFLICT OF INTEREST STATEMENT

The opinions and assertions herein are the private views of the authors and are not to be construed as official or as reflecting the views of the U.S. Army Medical Research and Development Command, the U.S. Army, or the U.S. Department of Defense. The authors declare no conflict of interest.

## ETHICS STATEMENT

All participants provided written informed consent. This study was conducted in accordance with the Declaration of Helsinki and received approval from the Internal Review Board of the University of Connecticut under reference H21‐0108 and BRANY IRB under reference B2023‐0039. This study was also reviewed and approved by DoD HRPO under E02882.1a.

## Data Availability

Source data for this study are not publicly available due to privacy or ethical restrictions. The source data are available to verified researchers upon request by contacting the PI, ECL.

## References

[phy270745-bib-0001] Akavian, I. , Epstein, Y. , Rabotin, A. , Peretz, S. , Charkoudian, N. , & Ketko, I. (2025). The significance of body surface area to mass ratio for thermal responses to a standardized exercise‐heat stress test. Medicine and Science in Sports and Exercise, 57, 88–93. 39207822 10.1249/MSS.0000000000003545

[phy270745-bib-0002] Alele, F. O. , Malau‐Aduli, B. S. , Malau‐Aduli, A. E. , & Crowe, M. J. (2021b). Individual anthropometric, aerobic capacity and demographic characteristics as predictors of heat intolerance in military populations. Medicina, 57, 173. 33671414 10.3390/medicina57020173 PMC7922340

[phy270745-bib-0003] Alele, F. O. , Malau‐Aduli, B. S. , Malau‐Aduli, A. E. O. , & Crowe, M. J. (2021a). Haematological, biochemical and hormonal biomarkers of heat intolerance in military personnel. Biology, 10, 1068. 34681165 10.3390/biology10101068 PMC8533107

[phy270745-bib-0004] Allaire, J. (2012). RStudio: Integrated Development Environment for R. Boston, MA, 770, 165–171.

[phy270745-bib-0005] Armstrong, L. E. , Maresh, C. M. , Castellani, J. W. , Bergeron, M. F. , Kenefick, R. W. , LaGasse, K. E. , & Riebe, D. (1994). Urinary indices of hydration status. International Journal of Sport Nutrition and Exercise Metabolism, 4, 265–279. 10.1123/ijsn.4.3.265 7987361

[phy270745-bib-0006] Borchers, H. W. , & Borchers, M. H. W. (2019). Package ‘pracma’. Practical numerical math functions, version 2.

[phy270745-bib-0007] Bouchama, A. , Abuyassin, B. , Lehe, C. , Laitano, O. , Jay, O. , O'Connor, F. G. , & Leon, L. R. (2022). Classic and exertional heatstroke. Nature Reviews Disease Primers, 8, 8. 10.1038/s41572-021-00334-6 35115565

[phy270745-bib-0008] Bowie, J. S. (2024). Physiological Modeling and Machine Learning in Exercise‐heat Acclimation of Men and Women. University of Connecticut.

[phy270745-bib-0009] Butler, C. , Dierickx, E. , Bruneau, M. , Stearns, R. , & Casa, D. J. (2023). Current clinical concepts: Heat tolerance testing. Journal of Athletic Training, 58, 84–90. 34793593 10.4085/1062-6050-352-21 PMC10072087

[phy270745-bib-0010] Casa, D. J. , Almquist, J. , Anderson, S. A. , Baker, L. , Bergeron, M. F. , Biagioli, B. , Boden, B. , Brenner, J. S. , Carroll, M. , & Colgate, B. (2013). The inter‐association task force for preventing sudden death in secondary school athletics programs: Best‐practices recommendations. Journal of Athletic Training, 48, 546–553. 23742253 10.4085/1062-6050-48.4.12 PMC3718357

[phy270745-bib-0011] Casa, D. J. , Armstrong, L. E. , Hillman, S. K. , Montain, S. J. , Reiff, R. V. , Rich, B. S. , & Roberts, W. O. (2000). National Athletic Trainers' association position statement: Fluid replacement for athletes. Journal of Athletic Training, 35, 212–224. 16558633 PMC1323420

[phy270745-bib-0012] Casa, D. J. , Armstrong, L. E. , Kenny, G. P. , O'Connor, F. G. , & Huggins, R. A. (2012). Exertional heat stroke: New concepts regarding cause and care. Current Sports Medicine Reports, 11, 115–123. 22580488 10.1249/JSR.0b013e31825615cc

[phy270745-bib-0013] Casa, D. J. , DeMartini, J. K. , Bergeron, M. F. , Csillan, D. , Eichner, E. R. , Lopez, R. M. , Ferrara, M. S. , Miller, K. C. , O'Connor, F. , & Sawka, M. N. (2015). National Athletic Trainers' Association position statement: Exertional heat illnesses. Journal of Athletic Training, 50, 986–1000. 26381473 10.4085/1062-6050-50.9.07 PMC4639891

[phy270745-bib-0014] Cohen, J. (1988). Statistical Power Analysis for the Behavioral Sciences. Routledge.

[phy270745-bib-0015] Cramer, M. N. , & Jay, O. (2019). Partitional calorimetry. Journal of Applied Physiology, 126, 267–277. 30496710 10.1152/japplphysiol.00191.2018 PMC6397408

[phy270745-bib-0016] Dreosti, A. (1935). Symposium‐problems arising out of temperature and humidity in deep mining on the Witwatersrand: The results of some investigations into the medical aspect of deep mining on the Witwatersrand. Journal of the Southern African Institute of Mining and Metallurgy, 36, 102–129.

[phy270745-bib-0017] Druyan, A. , Ketko, I. , Yanovich, R. , Epstein, Y. , & Heled, Y. (2013). Refining the distinction between heat tolerant and intolerant individuals during a heat tolerance test. Journal of Thermal Biology, 38, 539–542.

[phy270745-bib-0018] Druyan, A. , Makranz, C. , Moran, D. , Yanovich, R. , Epstein, Y. , & Heled, Y. (2012). Heat tolerance in women—Reconsidering the criteria. Aviation, Space, and Environmental Medicine, 83, 58–60. 22272518 10.3357/asem.3130.2012

[phy270745-bib-0019] Giersch, G. E. W. , Morrissey, M. C. , Katch, R. K. , Colburn, A. T. , Sims, S. T. , Stachenfeld, N. S. , & Casa, D. J. (2020). Menstrual cycle and thermoregulation during exercise in the heat: A systematic review and meta‐analysis. Journal of Science and Medicine in Sport, 23, 1134–1140. 32499153 10.1016/j.jsams.2020.05.014

[phy270745-bib-0020] Green, G. K. , Stone, W. J. , Tolusso, D. V. , Schafer, M. A. , & Lyons, T. S. (2023). A VO(2max) protocol for young, apparently healthy adults. International Journal of Exercise Science, 16, 1257–1268. 38288075 10.70252/MWVS5696 PMC10824284

[phy270745-bib-0021] Heaney, J. H. (2017). Components of an effective heat tolerance test for military populations. Journal of Science and Medicine in Sport, 20, S57.28928023

[phy270745-bib-0022] Heaney, J. H. , Hascall, J. L. , Wong, J. M. , Johnson, E. C. , & Miller, P. W. (2009). Use of a heat tolerance test to evaluate return to duty status in US Navy and Marine Corps personnel. In: 13th International Conference of Environmental Ergonomics.

[phy270745-bib-0023] Hedges, L. V. , & Olkin, I. (1985). Statistical Methods for Meta‐Analysis. Academic Press.

[phy270745-bib-0024] Howe, A. S. , & Boden, B. P. (2007). Heat‐related illness in athletes. The American Journal of Sports Medicine, 35, 1384–1395. 17609528 10.1177/0363546507305013

[phy270745-bib-0025] Kassambara, A. (2018). ggpubr: ‘ggplot2’ based publication ready plots. R package version 2.

[phy270745-bib-0026] Kazman, J. B. , Heled, Y. , Lisman, P. J. , Druyan, A. , Deuster, P. A. , & O'Connor, F. G. (2013). Exertional heat illness: The role of heat tolerance testing. Current Sports Medicine Reports, 12, 101–105. 23478560 10.1249/JSR.0b013e3182874d27

[phy270745-bib-0027] Kester, R. M. , Abraham, P. A. , Leggit, J. C. , Harp, J. B. , Kazman, J. B. , Deuster, P. A. , & O'Connor, F. G. (2024). Heat tolerance testing and the return to duty decision: A two‐year case cohort analysis. Journal of Special Operations Medicine: A Peer Reviewed Journal for SOF Medical Professionals W7TV‐MBRZ, 24, 48–52. 10.55460/W7TV-MBRZ 38360027

[phy270745-bib-0028] Ketko, I. , Eliyahu, U. , Epstein, Y. , & Heled, Y. (2014). The thermal‐circulatory ratio (TCR) an index to evaluate the tolerance to heat. Temperature, 1, 101–106. 10.4161/temp.29752 PMC4977162 27583291

[phy270745-bib-0029] Kolde, R. , & Kolde, M. R. (2015). Package ‘pheatmap’. R package 1: 790.

[phy270745-bib-0030] Koo, C. J. , Hintz, C. , & Butler, C. R. (2023). Return to duty following exertional heat stroke: A review. Military Medicine, 189, e1312–e1317. 10.1093/milmed/usad388 37776525

[phy270745-bib-0031] Lawrence, M. A. , & Lawrence, M. M. A. (2016). Package ‘ez’. R package version 4.

[phy270745-bib-0032] Lisman, P. , Kazman, J. B. , O'Connor, F. G. , Heled, Y. , & Deuster, P. A. (2014). Heat tolerance testing: Association between heat intolerance and anthropometric and fitness measurements. Military Medicine, 179, 1339–1346. 25373064 10.7205/MILMED-D-14-00169

[phy270745-bib-0033] Mann, T. , Lamberts, R. P. , & Lambert, M. I. (2013). Methods of prescribing relative exercise intensity: Physiological and practical considerations. Sports Medicine, 43, 613–625. 23620244 10.1007/s40279-013-0045-x

[phy270745-bib-0034] Mitchell, K. M. , Salgado, R. M. , Bradbury, K. E. , Charkoudian, N. , Kenefick, R. W. , & Cheuvront, S. N. (2021). Heat acclimation improves heat tolerance test specificity in a criteria‐dependent manner. Medicine and Science in Sports and Exercise, 53, 1050–1055. 33065595 10.1249/MSS.0000000000002545

[phy270745-bib-0035] Moran, D. S. , Erlich, T. , & Epstein, Y. (2007). The heat tolerance test: An efficient screening tool for evaluating susceptibility to heat. Journal of Sport Rehabilitation, 16, 215–221. 17923727 10.1123/jsr.16.3.215

[phy270745-bib-0036] Moran, D. S. , Heled, Y. , Still, L. , Laor, A. , & Shapiro, Y. (2004). Assessment of heat tolerance for post exertional heat stroke individuals. Medical Science Monitor: International Medical Journal of Experimental and Clinical Research, 10, CR252–CR257. 15173669

[phy270745-bib-0037] Mosteller, R. (1987). Simplified calculation of body‐surface area. The New England Journal of Medicine, 317, 1098. 3657876 10.1056/NEJM198710223171717

[phy270745-bib-0038] Northway, S. , Jones, D. M. , & Buono, M. J. (2023). Sensitivity and specificity of using exercise heart rate in a thermoneutral environment to predict heat tolerance status. International Journal of Occupational Medicine and Environmental Health, 36, 192–200. 37199403 10.13075/ijomeh.1896.02065 PMC10464765

[phy270745-bib-0039] O'Connor, F. G. , Casa, D. J. , Bergeron, M. F. , Carter, I. I. I. R. , Deuster, P. , Heled, Y. , Kark, J. , Leon, L. , MCDermott, B. , & O'Brien, K. (2010). American College of Sports Medicine roundtable on exertional heat stroke‐return to duty/return to play: Conference proceedings. Current Sports Medicine Reports, 9, 314–321. 20827100 10.1249/JSR.0b013e3181f1d183

[phy270745-bib-0040] Ott, R. , & Longnecker, M. (2010). An Introduction to Statistical Methods and Data Analysis. Cengage Learning Inc.

[phy270745-bib-0041] Périard, J. D. , Racinais, S. , & Sawka, M. N. (2015). Adaptations and mechanisms of human heat acclimation: Applications for competitive athletes and sports. Scandinavian Journal of Medicine & Science in Sports, 25, 20–38. 25943654 10.1111/sms.12408

[phy270745-bib-0042] R Core Team . (2013). R: A language and environment for statistical computing.

[phy270745-bib-0043] Rivera‐Brown, A. M. , Correa, J. J. , & Micheo, W. F. (2023). Return‐to‐competition progression after exertional heat stroke in an adolescent runner: A case report. Journal of Athletic Training, 58, 349–354. 35622950 10.4085/1062-6050-0583.21 PMC11215635

[phy270745-bib-0044] Roberts, W. O. , Armstrong, L. E. , Sawka, M. N. , Yeargin, S. W. , Heled, Y. , & O'Connor, F. G. (2021). ACSM expert consensus statement on exertional heat illness: Recognition, management, and return to activity. Current Sports Medicine Reports, 20, 470–484. 34524191 10.1249/JSR.0000000000000878

[phy270745-bib-0045] Roberts, W. O. , Armstrong, L. E. , Sawka, M. N. , Yeargin, S. W. , Heled, Y. , & O'Connor, F. G. (2023). ACSM expert consensus statement on exertional heat illness: Recognition, management, and return to activity. Current Sports Medicine Reports, 22, 134–149. 37036463 10.1249/JSR.0000000000001058

[phy270745-bib-0046] Schermann, H. , Craig, E. , Yanovich, E. , Ketko, I. , Kalmanovich, G. , & Yanovich, R. (2018). Probability of heat intolerance: Standardized interpretation of heat‐tolerance testing results versus specialist judgment. Journal of Athletic Training, 53, 423–430. 29775421 10.4085/1062-6050-519-16 PMC5967286

[phy270745-bib-0047] Schermann, H. , Heled, Y. , Fleischmann, C. , Ketko, I. , Schiffmann, N. , Epstein, Y. , & Yanovich, R. (2018). The validity of the heat tolerance test in prediction of recurrent exertional heat illness events. Journal of Science and Medicine in Sport, 21, 549–552. 29066054 10.1016/j.jsams.2017.10.001

[phy270745-bib-0048] Schneider, S. M. (2016). Heat acclimation: Gold mines and genes. Temperature, 3, 527–538. 10.1080/23328940.2016.1240749 PMC5198811 28090556

[phy270745-bib-0049] Sekiguchi, Y. , Benjamin, C. L. , Manning, C. N. , Struder, J. F. , Armstrong, L. E. , Lee, E. C. , Huggins, R. A. , Stearns, R. L. , Distefano, L. J. , & Casa, D. J. (2022). Effects of heat acclimatization, heat acclimation, and intermittent exercise heat training on time‐trial performance. Sports Health, 14, 694–701. 34706597 10.1177/19417381211050643 PMC9460081

[phy270745-bib-0050] Wickham, H. (2011). ggplot2. Wiley Interdisciplinary Reviews: Computational Statistics, 3, 180–185.

[phy270745-bib-0051] Wickham, H. , Averick, M. , Bryan, J. , Chang, W. , McGowan, L. D. A. , François, R. , Grolemund, G. , Hayes, A. , Henry, L. , & Hester, J. (2019). Welcome to the Tidyverse. Journal of Open Source Software, 4, 1686.

[phy270745-bib-0052] Yanovich, R. , Ketko, I. , Muginshtein‐Simkovitch, J. , Yanovich, E. , Eliyahu, U. , Fleischmann, C. , Atias‐Varon, D. , Waissengrin, B. , Makranz, C. , & Heled, Y. (2019). Physiological differences between heat tolerant and heat intolerant young healthy women. Research Quarterly for Exercise and Sport, 90, 307–317. 31169467 10.1080/02701367.2019.1599799

